# Sleeping Sickness at the Crossroads

**DOI:** 10.3390/tropicalmed5020057

**Published:** 2020-04-08

**Authors:** Christian Burri

**Affiliations:** 1 Swiss Tropical and Public Health Institute, Socinstrasse 57, 4002 Basel, Switzerland; christian.burri@swisstph.ch; 2 University of Basel, Petersplatz 1, 4001 Basel, Switzerland

## 1. A Disease with Historical Dimension

Human African trypanosomiasis (HAT; sleeping sickness) is a disease with truly historic dimensions. Its maximum possible distribution corresponds to the range of tsetse flies, which covers an area of eight million km^2^ between 14° North and 20° South latitude on the African continent. Trypanosomes are very ancient parasites, which emerged around 380 million years ago and today are ubiquitous. Some salivarian forms began to transmit to mammals when tsetse flies emerged some 35 million years ago. The relatively late arrival of humans may explain why African game animals are tolerant towards most species of trypanosomes, but humans and most domestic animals are susceptible to certain species. Accounts of encounters with cachectic and sleep-affected people and the death of important authorities were already reported by Arabian travellers in the 12th to 14th century [1].

HAT likely had a limited impact on the local population until the slave traders and later the colonial forces arrived in Africa. The tsetse belt was extremely thinly populated (< 6 persons/km^2^) and people lived in very small, dispersed villages. Tsetse infested areas were avoided due to the annoyance by the flies and tales of witchcraft, the bush around the villages was cleared for protection against game animals and in some cultures there was an awareness about the danger of the tsetse flies to cattle [2].

From the beginning of the times of colonization onwards, HAT has had a tremendous impact on populations and societies, and the disease is very closely tied to the development of Africa. HAT hampered the colonization of the continent, since, unlike the Conquistadors in Latin America, all invaders in Africa faced dramatic logistical problems caused by the fact that horses are highly susceptible to trypanosomes [3]. The impact on trade (particularly slave trade) was recognized very early on. In 1792, the British physician Thomas Winterbottom described a disease in Sierra Leone he depicted as ‘negro lethargy’. He observed that slave shippers rejected those with swelling of the posterior cervical lymph nodes—a sign associated with HAT that is still known as Winterbottom’s sign. Therefore, it is not difficult to understand the manifold early attempts to control the disease. However, it was only in 1895 when Sir David Bruce reported that the tsetse fly was linked to cattle trypanosomiasis, and Dutton in 1902 (West Africa) and Castellani 1903 (East Africa) detected the causative agents of African sleeping sickness [4].

These findings coincided with the first reported major epidemic in East Africa in 1900, which devastated the Busoga focus at the Kenya–Uganda border and left about half a million people dead. The responsible subspecies is not entirely clear until today. It was earlier described to be *T. b. gambiense*, however, at that time, the more likely East African subspecies, *T. b. rhodesiense*, had not yet been described; clinically the descriptions rather support the hypothesis that the latter pathogen was responsible [4]. HAT had a similar impact on the population in the Belgian Congo, e.g., along the Mpoko river: in 1917, 79,000 people were counted in a census, in 1919, only 1,200 people were still in the area. The colonial powers were horrified by the speed at which their working force was dying. They ordered the displacement of the population from the shores of Lake Victoria and infected people were isolated in sleeping sickness camps. Similar actions were taken in the Belgian Congo [3]. These activities marked the start of incredible efforts of the colonial powers to control the disease, but particularly also of the vertical approach towards disease control, with special programs run in parallel with the public health system—which is now one of the challenges in the elimination “end game” with very few patients remaining requiring an integrated public health approach.

The detection of the causative agent, the mode of transmission and the first documented major epidemics coincided with the advent of modern pharmacology. It is, therefore, no surprise that there was an interest to find drugs against this disease. In 1908, the colonial powers, in a joint conference, decided to give drug development a high priority and in course, several molecules were developed.

## 2. The First Turning Point and a Colossal Failure

The history of the oldest drug still in use against HAT, suramin, is described in the context of the history in the article of Madeja et al., Not only is the drug still manufactured, but also the continued support of Bayer since the year 2000 is a major contributing factor allowing us to write about the elimination of HAT today.

The activity of inorganic arsenic-based compounds had already been recognized in the mid-19th century and the treatment of “nagana” (animal trypanosomiasis) was described by David Livingstone in 1848 and David Bruce in 1895. This knowledge led to the development of the first organo-arsenic compound Atoxyl^®^ in 1905 by Paul Ehrlich, who used trypanosomes as a model to screen molecules, but was mainly searching for drugs against syphilis. Wolferstan Thomas in Liverpool subsequently showed that Atoxyl^®^ was effective against *T. b. gambiense*. However, Atoxyl^®^, meaning ‘non-toxic’, caused severe adverse drug reactions particularly affecting the optic nerve; it was only active against early stage HAT and was followed by tryparsamide in 1919. Tryparsamide was developed in the USA and was the first drug to be active against the late stage, although not very active and also prone to extensive resistance development. Tryparsamide was also very toxic, with a dose dependent risk damaging the optic nerve. This is documented in a horrifying report from 1930 when a lieutenant of the French Army in Cameroon doubled the prescribed dose of tryparsamide to speed up the recovery of 800 patients. Two days later, all these patients were blind. This event prompted the Swiss chemist and physician Dr. Friedheim to investigate alternative drugs. The introduction of a triazine ring lead to the development of melarsen (disodium p-melaminyl-phenyl arsonate) in 1940, which proved to be a very efficient drug against *T. b. gambiense*, but was still very toxic. In 1944, Friedheim first described the trivalent arsenoxide form of melarsan, known as melarsenoxide. He reported its use in treatment of human sleeping sickness in 1948. The main achievement was the reduction of the duration of therapy to six weeks, with two weeks of seven daily injections of 1.5 mg/kg each, spaced by an interval of one month. In a further step, capping the arsenic in melarsen oxide with British anti-Lewisite, an antidote to the arsenical warfare agent Lewisite, reduced the toxicity by a factor of the order of 100, but the trypanocidal activity only by a factor 2.5. The new drug was called melarsoprol (Mel B; Arsobal^®^) [5]. It remained the mainstay of second stage HAT treatment until the early 2000s despite the long treatment duration of 35 days, the known related adverse drug reactions, particularly the encephalopathic syndrome that occurs in about 10% of the patients treated and leads to their death in about 50% of cases, and the potential for drug resistance.

Another line of research was the molecules with a diamidine structure discovered in the 1930s. These molecules were detected by serendipity in the search for hypoglycaemic compounds, with the idea in mind that this effect might compromise the very prominent and particular glucose metabolism of trypanosomes. Several compounds, however, proved to have a direct trypanocidal effect with the three compounds stilbamidine, pentamidine, and propamidine identified to have the highest activity [6]. Pentamidine does not penetrate the blood brain barrier so its use is limited to first stage HAT; despite this drawback it is still is in use today, for treatment of children below six kilograms. The distinctly better safety profile of the drugs only active against first stage disease (pentamidine and suramin) versus melarsoprol was also the advent of the consistent performance of lumbar puncture to determine the disease stage and make a treatment decision. The need for a lumbar puncture was, for over 50 years, a characteristic of HAT treatment, a source of patient distress, stigma and technical limitation of treatment. Some 70 years after the discovery of pentamidine, the diamidines again became the focus of drug development, although not successful until today.

The development of the organo-arsenicals, diamidines, and also drugs developed much later and still in use like eflornithine and nifurtimox, including their drawbacks related to the potential for drug resistance are described in detail in the contribution of De Koning.

The “scramble for Africa” was an investment with very high political and economic stakes, and sleeping sickness was not just a disease; it had become the colonial disease. Besides drug development, the responses of the Anglophone and Francophone colonial powers to trypanosomiasis differed significantly. Francophone countries chose to concentrate directly on the medical problems presented by the disease in humans. This included the introduction of “mobile teams” actively searching and screening population for HAT cases. This method of systematic case detection and treatment with the aim of elimination of the parasite reservoir was suggested by the French military surgeon Eugène Jamot and such activities started in 1926 in Cameroon (“atoxylisation”) [7]. Subsequently, the prevalence of HAT declined from as high as 60% in 1919 to 0.2–4.1% in 1930, leading to an expansion of the methodology to other countries. After the Second World War, Atoxyl^®^ was replaced by pentamidine and a regular application of the drug every six months to the population at risk was introduced (“pentamidinisation”). In the 1950s in the Belgian Congo alone, some two million people were subjected to this preventive mass drug administration [3].

Due to the partial presence of *T. b. rhodesiense* in their territories, the Anglophone countries were confronted with the more widespread problem of disease in domestic livestock, which also presented a reservoir for human disease. Their approach included vector control (traps, spraying), bush clearing, and game destruction [3,7], and later the chemopreventive use of veterinary drugs (diminazene, isometamidium, and homidium).

The control measures were overall very successful and progressively controlled the disease, reaching a very low, generalized transmission by the mid-1960s, with a minimum of 4435 cases declared in Africa in 1964 [8]. However, the measures taken were very costly, but above all very unpopular in the communities. This led to concealment where possible, and made the longterm goal of elimination by chemotherapy difficult, if not impossible [3]. We could observe similar tendencies when conducting clinical trials in the 2000s; potential patients were mostly hiding away or fleeing the villages at the beginning of mobile team campaigns for reasons of fear of stigmatization, lumbar puncture, pain, and the possibility of being treated with the dangerous drug melarsoprol. The in-depth understanding of the communities’ beliefs, needs, and approaches is therefore key in a successful elimination attempt; insight on these topics are presented in the papers by and Falisse et al., Lee et al., and Palmer et al.

A factor mentioned by Winslow in 1951, which may still be under-researched today, is the relationship between the disease and poverty, particularly inadequate food supply, as the disease leads to unused land, which creates malnutrition [3]. Such a view would require an even more integrated approach towards disease control and elimination.

When the colonial powers withdrew from Africa between 1960 and 1975, a new era began. The young nations created their own institutions with the goal of continuing research towards the elimination of HAT (e.g., Kenya Trypanosomiasis Research Institute (KETRI), Nigerian Institute for Trypanosomiasis Research (NITR), Uganda Trypanosomiasis Research Organisation (UTRO), and Programme sur la Recherche sur la Trypanosomiase in Côte d’Ivoire (PRCT)). These institutions were reinforced since the 1970s by internationally funded institutes dedicated wholly or partly to trypanosomiasis research (e.g., International Laboratory for Research on Animal Diseases (ILRAD) in Kenya, International Centre of Insect Physiology and Ecology (ICIPE) in Kenya, International Trypanotolerance Centre (ITC) in the Gambia). However, in the course of monetary adjustments in the 1980s, the decreasing funds available, and the emergence or increase of other health priorities, institutions devoted to a single disease were no longer sustainable and they were continuously integrated or transformed into multilateral institutions. Overall, in the years after the independence process, the expenditures for HAT were reduced, and awareness and surveillance of the disease decreased. The number of mobile teams was decreased, and it was attempted to transfer activities to the public health system—without having the respective tools, approaches, and knowledge [9]. This, together with social instability, conflicts, and insecurity constraining disease control interventions led to a significant resurge of Gambiense HAT in the 1980s and 1990s [8], mainly affecting Angola, Congo, Southern Sudan, and the West Nile district of Uganda [1]. At the end of the 1990s, the situation was comparable to the one in the 1920 and 1930; the number of reported cases was almost 30,000 with 300,000 cases suspected [10].

## 3. The Second Turning Point—The Change for the Better

In 2001, we published a Special Issue in the Journal Tropical Medicine and International Health [11], asking ourselves whether there were new approaches to roll back HAT. Not only was this the time of 300,000 HAT cases suspected in 19 countries of sub-Saharan Africa, it was also the time when the organo-arsenic drug melarsoprol was still the only treatment available for second stage HAT. Treatment with melarsoprol required around 35 days of hospitalization with numerous and very painful injections, very severe adverse drug reactions like an encephalopathic syndrome were common and the mortality rate under treatment was as high as 2–10%.

In those days, an oncologist tried to comfort us, saying that a 95% treatment success rate for a disease with an inevitably fatal course was fantastic. We did not share this view, and rather expressed a dream: to make HAT “an ordinary” disease, which follows the usual pattern “test, treat, track”—without the need of a lumbar puncture to make treatment decisions, without a high mortality rate under treatment, without the pain, without the stigma.

Elimination was a very far-fetched goal at this time, but there were some first positive signals that the dimensions of the problem and its impact on society and development were being recognized. The conclusions adopted by the International Scientific Committee of Trypanosomiasis Research and Control (ISCTRC) in 1999 reflected a new awareness of the disease. The African Union member states were urged to give highest priority ranking to African trypanosomiasis in their development programs, and it was recommended that urgent and particular attention should be given to surveillance and intervention in epidemic areas, to drug availability and resistance, and to the implementation of operational research to respond to the needs of control programs. At their meeting in Lomé in July 2000 the OAU Heads of State and Governments signed a declaration of intent to eradicate tsetse flies on the African continent—something that will likely not happen, but it was the turning point towards manifold activities which make us today work towards elimination of HAT as a public health problem of 2020 and the interruption of transmission by 2030.

At the same time, Médecins sans Frontières’ Access to Medicines Campaign were able to make a compelling case that society needed to rethink drug discovery paradigms for neglected diseases. Aventis (now Sanofi) was persuaded to repurpose and develop the failed anti-cancer drug eflornithine for use against HAT and to donate it at no cost to the WHO for distribution in Africa. Millions of dollars were also provided by Aventis/Sanofi to the WHO, who could now develop new screening and intervention programmes [9]. Bayer signed a similar contract with the WHO, a success story and joint effort which has been renewed by both companies until today, and which is one of the strong drivers in control and now elimination. In 2001, the Bill and Melinda Gates Foundation selected HAT to be one of the first diseases they targeted through the Consortium of Parasitic Drug Development (CPDD) and shortly thereafter, the Drugs for Neglected Diseases initiative (DNDi) was founded. The beginning of the century was truly an exciting time for neglected diseases and for HAT in particular. The changes and significant impact on funding were later summarized in the landmark publication “the new landscape of neglected disease drug development” [12].

The changed situation immediately led to the initiation of several large scale activities in drug development and the term “elimination” in the context of HAT was mentioned for the first time by Dr. Jannin, leading the anti-HAT efforts at the WHO in 2004 [13]. Drug development had been virtually dormant for about 50 years. Although the cultivation and test methods for drug screening for anti-trypanosomal drugs had been developed in the 1980s and 1990s [14], the money for pursing lead compounds in preclinical work, translational studies and large-scale trials was too scarce in these days. During the 1990s, some initial limited drug activities were carried out on shoestring budgets: eflornithine, which had initially been developed in the 1970s as a potential anti-cancer drug, was found to be active against second stage Gambiense HAT. This discovery in the 1980s was a scientific breakthrough [15] and eflornithine was shown to be much safer compared to melarsoprol. The drug received orphan drug status by the US Food and Drug Administration in 1990; however, production was stopped after a few years and only resumed after significant public and political pressure. Eflornithine, however, was only introduced for treatment in a limited number of centres by MSF in 2000, but not until 2006 by the National Sleeping Sickness Control programs because of its limited availability, the initial high costs, and particularly the logistical challenge to transport the drug and its associated 56 bottles of sterile water per treatment. The turning point was when WHO launched a kit format and coordinated training of staff from National Sleeping Sickness Control Programs [16]. Furthermore, in the 1990s nifurtimox, developed against Chagas disease was used in experimental settings mainly to treat melarsoprol refractory cases [17].

In the mid-1990s, the pharmacokinetics of melarsoprol was elucidated. The assessment of a subsequently proposed abridged 10 days regimen in a large scale trial with 550 patients in Angola (Impamel I) allowed the replacement of the empirically derived complex schemes lasting from 25–36 days in 2003 [18,19,20]. Whereas the new regimen had major socio-economic advantages, the disappointment was that the frequency of the worst adverse drug reaction, the encephalopathic syndrome, remained at levels of 5–10% of patients treated, still resulting in death in 10–50% of those in whom encephalopathy developed. The metabolism of melarsoprol was elucidated somewhat later [21]. The finding that the major metabolite melarsenoxide covalently bound to a midsize protein triggered another large-scale clinical trial, which led to the elucidation of the nature of the encephalopathic syndrome. For several reasons, these data by Seixas et al. were so far only published in the form of a thesis, and are now presented in this issue.

The Impamel program (Improved Application of Melarsoprol; financed by the Swiss Agency for Development and Cooperation) may not have been a breakthrough towards a new treatment against second stage HAT, but it comprised the first large scale clinical trial on this disease executed according to Good Clinical Practice, and it demonstrated the feasibility of modern clinical development for neglected diseases under the challenging conditions in countries of Central Africa.

The early 2000s were dominated by the development of the oral prodrug pafuramidine against first stage HAT; the program failed at a late point of development, but it contributed much to the understanding of HAT chemotherapy and the conduct of clinical trials against HAT, which it is described in detail by the paper of Dickie et al.

In parallel, several trials assessing combinations of eflornithine, melarsoprol, and nifurtimox were conducted. In all trials, the efficacy was better in the combination arms compared to the monotherapies. However, combinations containing melarsoprol resulted in very high frequencies of severe adverse drug reactions and were rapidly abandoned [16]. A multiple-centre trial, conducted in the Republic of Congo and the Democratic Republic of the Congo (DRC) compared nifurtimox–eflornithine combination therapy (NECT) with the standard eflornithine therapy. NECT reduces the number of eflornithine infusions from 56 to 14, the total amount of eflornithine by half and the hospitalization time by one-third [22]. Based on the favourable results of the trials conducted, NECT was included for treatment of second stage Gambiense HAT into the WHO’s Essential Medicines List in 2009 [23], and for children in 2013 [16]. NECT can be considered a milestone improvement: under optimal conditions, fatality during treatment is 0.5% compared to 5–6% under melarsoprol [24]. The complexity of its application still restricts the use to the second stage disease, meaning that the lumbar puncture for diagnostic staging is still required [24], continuing until today.

To identify better alternatives, the Drugs for Neglected Diseases Initiative initiated a major compound mining effort in 2005 to explore new and old nitroimidazoles as drug leads against human African trypanosomiasis. One of the 830 compounds screened, fexinidazole, proved to be orally active against *T. b. gambiense* and *T. b. rhodesiense* in animal studies and had an excellent safety profile.

The development of this orally active compound is described in detail in the papers of Neau et al. and Dickie et al. Fexinidazole received a positive scientific opinion from the European Medicines Agency for treatment of Gambiense HAT in late 2018, it was approved by the drug regulatory authority of the DRC and added to the WHO list of essential medicines in 2019, and the first official application in the DRC happened at the end of January 2020 on World NTD day in a public ceremony. This deliberate coincidence of the date depicts the new integrated thinking of HAT control and elimination in the framework of NTDs clearly.

Fexinidazole will be an essential component towards HAT elimination. However, it has some limitations, which will hamper its widespread use in the field: its absorption is dependent on simultaneous food intake, or else only subtherapeutic drug levels are reached; based on the observation of a lowered efficacy in patients with advanced disease, a lumbar puncture for staging still is necessary in such patients; and the drug has not been tested yet for children below six years [25].

Hence, the search for “the magic bullet” [26] continues—with an excellent starting position compared to 20 years ago: for the first time in history, we can speak of a modest pipeline of anti-HAT drugs. One most promising candidate is in late clinical development, several compounds are well advanced in pre-clinical stages, and medicinal chemistry and lead selection work is continued as described in the contributions of Buckner et al., Kariuku et al., Lim et al., and Rao et al.

Currently, the leading novel class of molecules are the boron-containing benzoxaboroles. One candidate, SCYX-7158, acoziborole, entered Phase II/III assessment in 2016 [27]. The compound is described in the publication of Dickie et al. Should the development program be successful, acoziborole would further revolutionize the efforts to eliminate and sustain elimination of HAT. Due to its long half-life of 400 hours, it can be potentially used as a single-dose treatment and should it be well tolerated this would provide further options for decentralized use, and maybe even for “ring-treatment” of patient contacts following the example of ring-vaccinations used, e.g., in the control of the Ebola virus. With fexinidazole, and potentially even more with acoziborole, the focus will turn away from the discovery and development of better tools, to the understanding of the implementation, optimal use, including the needs and perception of patients.

The clinical research programs have contributed to the reduction of cases: new strong partnerships were formed as described by Taylor et al. and the conduct of clinical trials in a number of endemic areas per se has had an impact through staff training, attention to disease, and intensified active case search and treatment of a large number of patients as described by Mbo et al.

Besides the improvements of the renewed interest of governments and improved drug treatment, there are several other reasons for the decrease of HAT prevalence: the advances in diagnostics are one of the major factors. The serological card agglutination test for trypanosomes (CATT) first published in 1986 [28] had a paramount impact on how patients could be screened by mobile teams. The test was adapted and improved several times, and despite its disadvantages (insufficient specificity to confirm diagnosis, only available in larger batches, cold chain necessary), it has kept its place in HAT diagnosis. The mini-anion exchange chromatography for trypanosomes (mAECT) which increases the sensitivity to detect the parasite in the blood significantly was already published in 1976 [29,30], however, only the increased funding available allowed its more consistent use and therefore detection of cases with low parasitemia. The introduction of rapid diagnostic tests is a true advancement, but also lacks the specificity needed to make a final treatment decision [31]. Additional tools were recently developed but so far only introduced to a limited extent into routine use (e.g., loop-mediated isothermal amplification (LAMP) [32,33]; immune trypanolysis test [34]), which will both play a role in the “end-game”. The question, however, is how such tests will be used in the future, and in what settings. The currently ongoing research program DiTECT-HAT is set up do exactly that: it seeks to validate the performance of diagnostic tools and algorithms for early and rapid diagnosis of Gambiense HAT for passive case detection, post-elimination monitoring, and for assessing the therapeutic response [35].

In addition to the optimization of the technical aspects, however, it is of paramount interest to know about local settings, preferences, and the loss of skills in areas with decreasing patient numbers. The paper by Palmer et al. reports on such investigations carried out in Uganda. Benhamou et al., through a case report on a repeatedly misdiagnosed patient, gives us an insight on future challenges for rapid diagnosis if knowledge and interest in the public health system is not maintained and broadened. Another unresolved caveat of diagnosis is that a number of patients are determined to be seropositive, but thereafter HAT cannot be confirmed. It will be one of the leading discussions when defining future strategies, and what to do in such cases. Nkieri et al. investigated the extent of this phenomenon in the still affected regions of DRC.

On one hand, relapses have always been a major challenge in the treatment of HAT and have made follow-up periods of up 24 months after treatment necessary [23]. On the other hand, until a few years ago, the dogma was “infected, but not treated inevitably leads to the death of the patient”. Reports on patients surviving for longer periods despite infection with trypanosomes emerged in the past few years [36]. One of the compartments where trypanosome may survive seems to be the skin [37,38]. This might also explain how HAT can re-emerge in so-called silent foci as illustrated by a nine-year-old child, who was diagnosed with Gambiense-HAT in Ghana in 2013, 10 years after the last detected case [39]. In this light, the findings of Mudji et al. also have importance: over ten years after treatment in the framework of clinical trials, a number of patients revisited presented continued signs and symptoms seen in HAT (lymphadenopathy, severe headaches, sleep disturbances); since no trypanosomes could be identified by any means, the implication of these findings remain open at this point.

In any case, the existence of such long-term cases has a sudden and dramatic impact on the view of HAT epidemiology and HAT elimination [39].

Besides further epidemiological, parasitological, and molecular research, mathematical modelling may help to improve our epidemiological knowledge and inform about elimination strategies [40] and their related costs [41]. This field has significantly developed against all odds in the past years: trypanosomiasis with its extremely focal distribution and the many external factors influencing its transmission has been a true headache over two decades for all modellers and predictive mappers. Studies of existing Gambiense-HAT models in a few foci (i.e., DRC, Guinea, and Chad) suggest that some type of additional infection reservoir is needed to match the observed dynamics of reported HAT cases [42,43]. This could arise from another human reservoir (including undiagnosed and latent infections), an animal reservoir, and/or heterogeneities in human risk exposure and surveillance coverage [39].

The French colonial forces had completely dismissed the value of vector control due the successes of the treatment strategy proposed by Laveran. However, vector control may play a larger role in Gambiense HAT elimination than anticipated. Historical investigations, practical intervention studies, and modelling demonstrate the significant role that vector control can play in the control of Gambiense HAT. Recent models suggest vector control will be essential if we are to reach the set target of elimination of the diseases as a public health problem by 2020 and beyond [44,45]. The fact that neither modelling nor vector control are represented in this edition does not represent a valuation of these topics.

## 4. Towards Elimination or a Dreadful Comeback?

This Special Issue comes in a very timely moment, because it is now important to secure what has been achieved, to understand missing pieces, and to finish the work. However, several challenges have to be overcome to not to end up, again, in disaster. In 2012, the World Health Organization, which has played an instrumental role in the control, set the goal for the elimination of human African trypanosomiasis (HAT), caused by *Trypanosoma brucei gambiense* (gHAT), as a public health problem for 2020 and for the total interruption of transmission to humans for 2030. The efforts to maximise output and optimize innovation by the WHO has intensified since, and several stakeholders and expert groups have been created and convened [46]. Since 2012, the spectacular decrease of the case number has continued: some 2,164 cases were reported in 2016, far fewer than the targeted 2016 milestone of 4000 cases, and 660 in 2018 [47,48].

First of all, “donor fatigue” must be avoided. The elimination to “no transmission” in the DRC where over 95% of the cases are nowadays occurring is a Herculean task, which will not happen without considerable and continued funding. The conventional measurements of success (e.g., US$ spent per DALY prevented) inevitably fail in an elimination scenario. Naturally, the amount of money spent per patient identified and treated will soar, so the question to the health economists is, rather how much money do we lose in case efforts would not be continued, factoring in the needed future efforts to re-start and control the disease again. Decisions on priorities will be necessary, too: whereas the total number of patients has massively decreased, the area in the DRC they are coming from has not. Therefore, a vast area still has to be kept under surveillance; this area has to be gradually reduced by safely “closing” focus by focus in order to not jeopardize the efforts. The elimination of HAT, malaria, and Guinea worm were all believed at a certain point to only be a matter of time—before new reservoirs became known (Guinea worm), pestidicide, and drug resistance set in (malaria), and interest was decreased in a premature belief in success (malaria and HAT).

Secondly, the human factor will start to play a key role: in theory, fexinidazole could be applied in 1,338 fixed health facilities (2017) an increase up by 52% from 2015 [47], and it will be many more, should acoziborole make it to application in a few years. However, HAT is a massively stigmatized disease, linked to many beliefs and bad spirits. Traditionally, patients after treatment were excluded from working and sexual intercourse for six months [49]. Therefore, the questions are: “will the disease be recognized by the younger physicians and nurses who have never seen a case of HAT?” “Is the medical staff willing to recognize a suspected case, given this will create a massive workload including trouble with the relatives of the patient and village, paperwork, an invasion of specialists for diagnosis and follow up activities?” “Is the medical staff that was told for over 100 years that HAT belongs into the specialized hands of the vertical programs willing to assume this task and challenge?” “Are the patients willing to accept HAT as a diagnosis anymore?” “Can we overcome wrong dogma and information?” and “Will patients falling into the respective category accept a lumbar puncture?” These questions can only be addressed through the thorough understanding of beliefs, perceptions, preferences, and decision-making processes. Therefore, the social and anthropological science, as well as health economics, will start to play a key role in the “end game”. The articles presented in this issue by Falisse et al., and Lee et al. are contributing to this area.

Thirdly, peace, stability, and a minimal standard of living for the people in the remote regions most affected are necessary to achieve disease elimination—this condition has not yet been met. There is little the scientific community can directly contribute to this—however, knowing that disease and poverty are inextricably linked [50], our efforts have to continue.

Finally, Rhodesiense HAT (the East African form of the disease) was reduced to as little as 54 cases in 2016 [47], goals have been reached and it may be seen as a quantité négligable. However, the disease with a zoonotic reservoir has the potential for spectacular returns, and this real danger still exists as described in the contribution of Matovu et al. The surveillance and the knowledge of local medical staff has dwindled, innovation was absent for *T. b. rhodesiense* for decades, and serological instead of microscopic tests are used for diagnosis of other diseases making accidental diagnosis impossible. From January to October 2019, a total of 2–8 cases were reported per month from all treatment centres in Malawi; in November 2019 to January 2020, this number surged to 25 and higher. The cases were reported from populations around the two geographically separate wildlife reserve areas, Vwaza and Nkhotakota; the reason for this increase is, so far, unknown (personal communication, World Health Organization, Control of Neglected Tropical Diseases, Geneva). This outbreak causes major concern and should be a serious warning to everyone who is of the opinion that sleeping sickness has been conquered. Similarly, other unexpected priorities, such as the current SARS-CoV-2 epidemic, may at all times derail a fragile health system. As soon as the mental and financial attention and priority is on another disease, signals of a HAT resurgence may well be overlooked—we should now be well aware about the consequences and impact of late reactions and exponential transmission.

Compared to when I wrote the conclusion of the 2001 HAT Special Issue in [11], we are at a completely different point today. We celebrated several marvellous scientific successes in the meantime, tools were improved, patients numbers down—but to reach the set goals and to get completely rid of this horrible disease, the conclusion is the same again: “The goal must now be to maintain the momentum” and “even the biggest efforts of the research scientists, field workers, development agencies, and companies will fail if they are not paralleled by achievements in the political field bringing peace, stability, and a minimal standard of living to the people in the remote regions most affected”.
